# Clonal complexes and virulence factors of *Staphylococcus aureus* from several cities in India

**DOI:** 10.1186/1471-2180-12-64

**Published:** 2012-05-01

**Authors:** Srikanth Shambat, Savitha Nadig, Sushma Prabhakara, Michele Bes, Jerome Etienne, Gayathri Arakere

**Affiliations:** 1 Society for Innovation and Development, Department of Microbiology, Indian Institute of Science, Bengaluru 560012, India; 2 Karolinska Institutet, Center for Infectious Medicine F59, Karolinska University Hospital, Huddinge, S-141 86 Stockholm, Sweden; 3 French National Reference Centre for Staphylococci, Hospices Civils de Lyon, Lyon, France; 4 University of Lyon, Inserm U851, Faculte de Medicine Lyon Est, Lyon, France

## Abstract

**Background:**

Diseases from *Staphylococcus aureus* are a major problem in Indian hospitals and recent studies point to infiltration of community associated methicillin resistant *S. aureus* (CA-MRSA) into hospitals. Although CA-MRSA are genetically different from nosocomial MRSA, the distinction between the two groups is blurring as CA-MRSA are showing multidrug resistance and are endemic in many hospitals. Our survey of samples collected from Indian hospitals between 2004 and 2006 had shown mainly hospital associated methicillin resistant *Staphylococcus aureus* (HA-MRSA) carrying staphylococcal cassette chromosome *mec* (SCC*mec*) type III and IIIA. But *S. aureus* isolates collected from 2007 onwards from community and hospital settings in India have shown SCC*mec* type IV and V cassettes while several variations of type IV SCC*mec* cassettes from IVa to IVj have been found in other parts of the world. In the present study, we have collected nasal swabs from rural and urban healthy carriers and pus, blood etc from in patients from hospitals to study the distribution of SCC*mec* elements and sequence types (STs) in the community and hospital environment. We performed molecular characterization of all the isolates to determine their lineage and microarray of select isolates from each sequence type to analyze their toxins, virulence and immune-evasion factors.

**Results:**

Molecular analyses of 68 *S. aureus* isolates from in and around Bengaluru and three other Indian cities have been carried out. The chosen isolates fall into fifteen STs with all major clonal complexes (CC) present along with some minor ones. The dominant MRSA clones are ST22 and ST772 among healthy carriers and patients. We are reporting three novel clones, two methicillin sensitive *S. aureus* (MSSA) isolates belonging to ST291 (related to ST398 which is live stock associated), and two MRSA clones, ST1208 (CC8), and ST672 as emerging clones in this study for the first time. Sixty nine percent of isolates carry Panton- Valentine Leucocidin genes (*PVL*) along with many other toxins. There is more diversity of STs among methicillin sensitive *S. aureus* than resistant ones. Microarray analysis of isolates belonging to different STs gives an insight into major toxins, virulence factors, adhesion and immune evasion factors present among the isolates in various parts of India.

**Conclusions:**

*S. aureus* isolates reported in this study belong to a highly diverse group of STs and CC and we are reporting several new STs which have not been reported earlier along with factors influencing virulence and host pathogen interactions.

## Background

*S. aureus* is a highly versatile gram positive organism capable of being a commensal and causing a variety of diseases such as soft tissue infections, bacterial endocarditis, septicemia and osteomyelitis. The ability of the organism to cause a multitude of infections is probably due to the expression of myriads of different toxins, virulence factors and also cell wall adhesion proteins and staphylococcal superantigen like proteins (ssl) involved in immune-evasion. The emergence of MRSA in most countries of the world is a cause of great concern. Vancomycin resistance, in addition, has left physicians with limited treatment options [1,2].

The distinction between HA- MRSA and CA- MRSA was clear when CA-MRSA were first reported. CA-MRSA originated with individuals in the community who had none of the risk factors from exposure to hospital environment and had distinctly different antibiotic sensitivities than the HA-MRSA which infected hospitalized patients with specific risks of infections. But in the last five years, CA-MRSA have infiltrated the hospitals and are replacing HA-MRSA, mainly in countries where the prevalence of CA-MRSA is high [3].

 Methicillin resistance is conferred on the organism by the presence of a unique mobile genetic element called the SCC*mec* carrying the *mecA* gene. The SCC*mec* elements are divided into different types based on the nucleotide differences in two essential components, *ccr* (cassette chromosome recombinase) gene complex, represented by *ccr* genes and *mec* gene complexes. Eight major types of SCC*mec* elements were reported till recently but three more new types have been added in the past few months from bovine and human origins increasing the total to eleven SCC*mec* types [4-6]. HA-MRSA isolates contain mainly type I, II, and III SCC*mec* elements while CA-MRSA contain type IV and V SCC*mec* elements each of which has several variants. For instance, majority of Indian HA-MRSA collected between 2002 and 2006 contained type III or IIIA SCC*mec* elements, as previously reported [7,8].

We reported in 2008 the presence of *PVL* positive ST22 (EMRSA-15) and ST772 (single locus variant of ST1 and belonging to CC1) as major clones in nasal swabs collected in healthy carriers in and around Bengaluru in a small number of samples [9]. Recently, our studies in carriers and individuals with disease from rural and urban areas of Bengaluru showed variants of EMRSA-15 clones [10]. Another study from a tertiary care hospital in Mumbai also demonstrated the presence of EMRSA-15 as a major clone among patients [11]. A second important clone is the *PVL*-positive ST772 which has been detected in and around Bengaluru, Mumbai (India), Bangladesh and Malaysia [9,11-13]. These two dominant epidemic clones are associated with both CA- and HA-infections in India and seem to have progressively replaced the ST239 clone in hospitals [11].

Aim of this study was to establish the lineage of sensitive and resistant *S. aureus* strains collected from in and around Bengaluru and three other cities in India, and determine their toxins and virulence factors. In this article, MRSA and MSSA collected either from HA- and CA-infections or carriages were characterized using the microarray system developed by Clonediag® which detects 300 alleles of the *S. aureus* genome [14]. This characterization complemented those obtained by multi-locus sequence typing (MLST), staphylococcal protein A (*spa)* typing, pulsed field gel electrophoresis (PFGE), PCR to confirm the SCC*mec* type, toxin gene content, and antibiograms. The two already-reported ST22 and ST772 clones were detected as MSSA and MRSA. The spreading of ST8 along with an emerging clone of PVL-negative ST672 among Indian CA-MRSA is being reported in this study. The Indian MSSA clones identified were much more diverse and were different from the MRSA clones, except for ST8 and 672 which were detected in both MRSA and MSSA groups. The livestock-associated ST398 related clone (ST291) is reported for the first time in two MSSA isolates.

## Results

### Carrier and disease *S. aureus* isolates

Carrier (38) and disease (30) isolates were collected from rural, urban out patient and urban in patient environments and analysis is presented in Table 1.

**Table 1:** Molecular characteristics of MSSA/MRSA clones from carriers and disease isolates

**CC/ST**	**N (%)**	**Carrier/Disease isolates N/N**	**MRSA N (%) {Carrier/Disease, N}**	**SCC*mec* typ*e***	***spa* types (MRSA/MSSA)**	***agr* type**	***PVL* genes N (%)**	***tst-1* N (%)**	***egc* N (%)**	**Other genes (N)**	**Capsular type**
CC22-ST22	19 (28)	8/11	13 (68)	IV	t852 (13/0)	I	19 (100)	0/19	19 (100)	*sec, sel* (1)	5
			4/9		t005 (0/5)					*sea, seb* (1)	
					t2986 (0/1)						
CC1-ST772	13 (19)	7/6	9 (69)	V	t657 (5 /1)	II	13 (100)	0	13 (100)	*sea, sec, sel* (5)	5
			4/5		**t3387**^1^ (2/0)					*sea, see* (3)	
					t1387 (1/0)					*sea* (3)	
					**t1839** (0/1)					*sea, seb* (1)	
					**t1998** (0/1)					*sea, sec, sel, see* (1)	
					**t3596** (1/0)						
					t345 (0/1)						
CC121-ST120	7 (10)	4/3	0		**t3204** (0/2)	IV	7 (100)	0	7 (100)	*sec (3), sea, seb,sec* (1)	8
					**t1999** (0/2)					*seb,sec* (1)	
					t159 (0/3)						
ST672	4 (6)	2/2	2 (50)	V	**t1309** (2/0)	I	0	0	4 (100)	*sea, seb* (1), *sea* (1)	8
			0/2		**t3840** (0/1)						
					**t3841** (0/1)						
CC45-ST45	4 (6)	3/1	0		t939 (0/1)	I ^2^	0	0	4 (100)	*sec, sel* (1)	8
					**t4074** (0/2)						
					**t3537** (0/1)						
CC5-ST5	4 (6)	4/0	0		t442 (0/3)	II	1 (25)	0	4 (100)	*sea, sed, ser* (1)	5
					**t3597** (0/1)					*see, sed, ser* (1)	
										*see* (1), *edinB* (1)	
CC8-ST1208	3 (4.4)	1/2	3 (100)	V	t064 (3/0)	I	1 (33)	0	0	*sea, seb, sek, seq,see* (2)	5
			1/2							*sea, seb, sek, seq* (1)	
ST72	1 (1.5)	1/0	0		t148 (0/1)	I	1 (100)	1 (100)	1 (100)	*sec, sel* (1)	5
CC30-ST30	4 (6)	1/3	1 (25)	IV	t021 (1/3)	III	4 (100)	0	4 (100)	*sea, seb* (2)	8
			1/0							*sea* (1)	
ST39	1 (1.5)	1/0	0		t096 (0/1)	III	0	0	0	*sea* (1)	
CC398-ST291	2 (3)	2/0	0		t937 (0/1)	I	0	0	1 (50)	*etd, edinB* (2)	5
					t3096 (0/1)						
CC15-ST199	2 (3)	0/2	0		t774 (0/2)	II	0	0	0	None	8
ST6	3 (4.4)	3/0	0		t304 (0/1)	I	0	1 (33)	0	*sea, sel (1)*	8
					**t4285** (0/1)					*sea, seb, sek, seq, see*(1)	
					t701 (0/1)					*sel* (1)	
ST7	1 (1)	1/0	0		t091 (0/1)	I	0	0	0	*sep*	8
**Total**	**68**	**38/30**	**28 (41)**				**47 (69)**		**57 (84)**		

^1^New spa types reported to the data base; ^2^ 1 isolate is *agr* negative.

Twenty six percent of carrier isolates and sixty percent of disease isolates were MRSA. All MRSA carried SCC*mec* type IV or V. Total of 15 STs were present among all the 68 isolates characterized. All but one sequence type were present in carrier isolates. ST 22, 772, 30, 121, 1208, 199, 672, and 45 were present among disease isolates. ST 5, 6, 7, 39, 72, and 291were present only among carriers. Antibiotic sensitivity to five antibiotics -oxacillin, cefoxitin, erythromycin, gentamicin, and tetracycline were tested on all the strains (data not presented). Isolates belonging exclusively to carrier STs were sensitive to all the antibiotics tested. Predominant methicillin resistant STs were 22 (68%) and 772 (69%) along with small percentage of isolates belonging to ST30, 672 and 1208 carrying 1.5, 3.0 and 4.4 percent of isolates respectively as MRSA. Carrier MRSA isolates were limited to ST22, 772, 30 and 1208 while disease MRSA isolates in addition included ST672. All carrier and disease isolates of ST22 and 772 lineage were *PVL* and *egc* positive.

### MLST types

**Figure 1:**
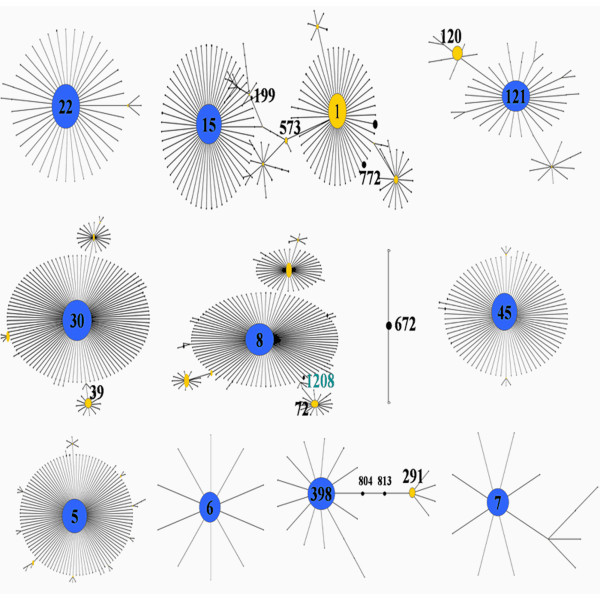
**eBURST analysis of 15 STs present among the Indian *Staphylococcus aureus* collection.**

Twelve *S. aureus* CC (15 STs) were identified with three of the clones detected in more than 10% of the isolates (ST22, ST772 and ST121) (Table 1). New or recently emerging clones were also detected (ST1208 and ST672). Figure 1 shows the eBURST analysis and lineages of all sequence types. Details of all the STs follow as given below. CC and STs of MSSA were much more diverse than those of MRSA (12 for MSSA, 5 for MRSA). Isolates belonged to all the 4 *agr* types. New *spa* types were detected among MRSA and MSSA isolates of lineages ST672, 772, 45, 121 and 6. *PVL* genes were detected in 69% of the isolates and *egc* in 84%. Microarray analysis was performed for representative carrier and disease isolates from each sequence type to determine the virulent factors and toxins.

### Microarray

Factors which were common to all isolates when analyzing the microarray results, were as follows: virulence factor genes- α, γ, δ haemolysins, staphylococcal complement inhibitor (*scn*), aureolysin, *sspA, sspB* and *sspP*; MSCRAMMS genes- *fnbA, fib, ebpS, vwb, sdrC*; Clumping factors A and B; *bbp* (bone sialo-protein binding protein); *map* (major histocompatibility complex class II analog protein) and immune-evasion genes- *isaB, isdA, imrP, mprF, hysA1, hysA2, set 6, ssl9* were present in all except in one isolate of ST199 and one isolate of ST22, *ssl*7 absent only in one isolate of ST121. The patterns of presence and absence of virulence and immune evasion factors strictly followed the sequence type. Carrier and disease isolates belonging to a particular ST type had the same patterns. Raw microarray data of 33 isolates is provided as an Additional file 1. In a few cases where results were ambiguous, results have been confirmed with PCRs.

### PFGE

**Figure 2:**
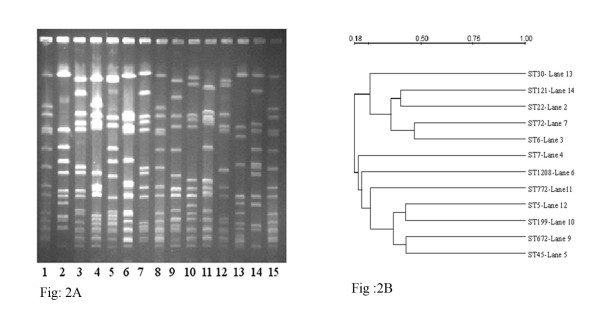
**A: PFGE patterns of *SmaI * digested isolates showing different sequence types of Indian *S. aureus.* ** Lane: 1, 8,15 - NCTC8325, Lane 2 - ST22, Lane 3 - ST6, Lane 4 – ST7, Lane 5 – ST45, Lane 6 – ST1208, Lane 7 – ST72, Lane 9 – ST672, Lane 10 – ST199, Lane 11 – ST772, Lane 12 – ST5, Lane 13 – ST30, Lane 14 – ST121. **B**: Dendrogram of PFGE based on similarities derived from the UPGMA and dice coefficients using Quantity one software.

Figure 2A represents PFGE patterns of one representative isolate from each ST and 2B the dendrogram of PFGE depicting the relatedness of patterns based on the similarities derived from the UPGMA and dice coefficients using the Quantity one software. All profiles were different from each other and were distinct patterns characteristic of the ST.

### CC22-ST22

ST22 is the major clone detected in 28% of the isolates present in both carrier and disease isolates. Methicillin resistance was detected in 68% in both groups, and the MRSA isolates had a SCC*mec* IV element. PFGE patterns of all ST22 isolates resembled classical EMRSA-15 patterns with 3–4 band differences and were related variants [10]. *Spa* types from MSSA isolates differed from those of MRSA. ST22 is the clone most resistant to antibiotics with resistance to gentamicin and erythromycin, in MRSA as well as MSSA, both in carriers and infected patients. This clone was *agr* type I, capsular type 5, *PVL* and *egc* positive.

### CC1-ST772

This is the second major clone present in our collection detected in 19% of the isolates both in carrier and disease isolates. Methicillin resistance was detected in 69% in both groups and the isolates had a SCC*mec* V element. Isolates with resistance to gentamicin and erythromycin were found in MRSA only, but both in carriers and infected patients. *Spa* types from MSSA isolates differed from MRSA. This clone was *agr* type II, capsular type 5, *PVL* and *egc* positive.

### CC121-ST120 and ST121

The ST120/121 clones were detected in 10% of the isolates both in carriers and patients. Methicillin resistance as well as resistance to other antibiotics was not detected in any of the isolates. This clone was *agr* type IV, capsular type 8, *PVL* and *egc* positive.

### ST672

We are reporting a new sequence type from India, which appears to have the potential to be a founder clone. This clone was detected in 6% of the isolates in both carrier and disease isolates. Methicillin and gentamicin resistance was detected in 2 disease isolates with a SCC*mec* V element. *Spa* types from MSSA isolates differed from those of MRSA. This clone was *agr* type I, capsular type 8, *PVL* negative and *egc and seb* positive.

### CC8-ST1208 and ST72

ST1208 is a new single locus variant (SLV) of ST8 and ST72 is a double locus variant (DLV). One ST1208 isolate was *PVL* positive. All three ST1208 MRSA isolates and one ST72 MSSA isolate were resistant to gentamicin and erythromycin. These clones were *agr* type I, and capsular polysaccharide type 5.

### CC30-ST30 and ST39

CC30 was represented by 4 isolates from the community and the hospitals belonging to ST30 and one ST39 carrier isolate (SLV of ST30). Methicillin and erythromycin resistance was detected in one ST30 carrier isolate with SCC*mec* type IVc. All isolates were *agr* type III. This is the only SCC*mec* type IVc isolate belonging to *agr* type III in our collection with a distinct PFGE pattern different from EMRSA-15. Except for one carrier ST39 MSSA isolate, all isolates were *PVL* and *egc* positive and belonged to capsular polysaccharide type 8.

### CC398-ST291

This is the first report of two carrier MSSA isolates which are related to *S. aureus* from bovine origin. ST291 is a DLV of ST398 and *spa* types t937 and t3096 differed by one repeat unit. No antibiotic resistance was detected. PFGE patterns of these two isolates were very closely related with one band difference. These two isolates contained exotoxin D (*etD*) and *edinB* (epidermal cell differentiation inhibitor B) unlike other isolates and were negative for *PVL* and *tst* and contained capsular polysaccharide type 5.

### CC45-ST45, CC5-ST5, CC15-ST199, ST6 and ST7

These five other STs included 14 isolates with various characteristics. Methicillin resistant isolates were not detected among these STs, as well as other antibiotic resistance determinants. The *PVL* genes were detected in two isolates. While ST6, 7, 45, and 199 had capsular polysaccharide type 8, CC5 contained type 5.

### Differences in SCC*mec* elements of MRSA isolates

Table 2 represents the PCR and microarray data for all MRSA (A) and representative carrier and disease isolates belonging to SCC*mec* type IV and V (B and C) respectively. After determination of *mecA* gene in all 68 samples, multiplex PCRs were performed for determination of the *mec* and *ccr* complexes using primers for amplification of *ΔmecR1, IS1272, dcs, ccrA2B2, ccrC*, *mec* C2 complex, subtypes of SCC*mec* type IV from IVa to IVd and IVh only for MRSA isolates. Various regions of SCC*mec* type V element from known sequences were also amplified by PCR to further identify SCCmec type V isolates.

**Table 2:** **Characteristics of representative SCC *mec* type IV and V isolates examined by PCR and Microarray**

**A PCR**	**ST/# isolates**	** *mec A* **	** *Δmec R* **	** *ccr A2* **	** *ccr B2* **	** *dcs* **	** *IS 1272* **	** *ccrC* **	** *mecC2. com* **	** *PVL* **	** *hsd S* **	** *hsdR* **	** *hsdM* **
	**22/13**	+	+	+	+	+	+	-	-	+	-	-	-
	**772/9**	+	-	-	-	-	-	+	+	+	-	+	+
	**1208/3**	+	-	-	-	-	-	+	+	-	-	-	+
	**672/2**	+	-	-	-	-	-	+	+	-	-	+	+
**B PCR + MA**	**ST/# isolates**	** *mecA* **	** *ΔmecR* **	** *Ccr A2* **	** *ccr B2* **	** *ccrC* **	** *PVL* **	** *Agr type* **	** *sea* **	** *seb* **	** *sec* **	** *sed* **	** *see* **
	**22/5**	+	+	+	+	-	+	**I**	-	-	-	-	-
	**772/2**	+	-	-	-	+	+	**II**	+	-	+	-	-
	**1208/3**	+	-	-	-	+	-	**I**	+	+	-	-	-
	**672/1**	+	-	-	-	+	-	**I**	-	-	-	-	-
**C MA**	**ST/# isolates**	** *blaZ* **	** *blaI* **	** *blaR* **	** *hlb* **	** *sak* **	** *chip* **	** *scn* **	ccrC* (ZH47)	** *Egc* **	** *splA* **	** *splB* **	** *splE* **
	**22/5**	+	+	+	+	+	+	+	-	+	-	-	-
	**772/2**	+	+	+	-	-	-	+	+	+	-	-	-
	**1208/3**	+	+	+	+	+	-	+	-	-	+	+	+
	**672/1**	+	+	+	+	+	-	+	+	+	-	-	-

* Second *ccr C* present in SCC*mec*ZH47.

Isolates carrying SCC*mec* type IV cassettes did not amplify primers specific for IVa, IVb, IVc, IVd and IVh. Previous work from our laboratory has shown several variants of classical EMRSA-15 in PFGE patterns, and the J regions could be different from the known ST22, EMRSA-15 isolates [10]. One ST30 carrier isolate carrying SCC*mec* type IV has a different PFGE pattern from that of ST22 (Figure 2) and amplified primers specific for SCC*mec* type IVc.

### Differences in type V SCC*mec* elements

SCC*mec* type V elements were present in three different classes of STs-772, 672 and 1208. PCRs to identify different regions of type V elements (using strain WIS (WBG8318), Genbank accession no. AB121219) and microarray of selected isolates pointed to two different variants of type V element as shown in Table 2 (B and C). *CcrC, mecA* and *ugpQ* (Glycerophosphoryl-diester-Phosphodiesterase next to *mecA*) were present in all type V isolates while only isolates belonging to ST772 and ST672 carried a second *ccrC* region in the SCC*mec*ZH47 in the microarray from the mosaic cassette ZH47 reported by Heuser et al [15]. This region was positive by PCR using primers specific for the second *ccrC* in the SCC*mec*ZH47 region with a size of 435 bp and is identical in sequence to isolates containing composite cassettes of SCC*mec* type V (5&5 C2). Type V isolates belonging to CC8 did not carry the second *ccrC* region. SCC*mec*ZH47 also contain *ccrA2ccrB2* and a very small truncated *mecR* region which did not amplify in our ST772 and ST672 isolates by PCR and microarray. Apart from amplifying the *mec*C2 complex upstream of *mecA*, none of the primers designed for several different regions of SCC*mec* type V based on sequences from WIS strain, amplified DNA from our type V isolates indicating that the J regions could be different. All isolates belonging to ST672 and 772 amplified primers for both *hsdR* and *hsdM* regions while ST1208 isolates did not amplify the *hsdR* region indicating there could be changes in this region as well (Table 2A). No DNA fragments targeting hsdS, which determine the specificity of restriction modification system, were amplified with DNAs of all isolates. The other genes indicated in Table 2C are selected from the microarray data to examine the differences among isolates belonging to different STs.

## Discussion

We have characterized *S. aureus* isolates from different cities in India, which belong to a wide variety of STs from healthy carriers and individuals with simple to complicated diseases. Even in a small number of isolates (68), there were 15 different STs (including the two isolates resembling *S. aureus* from animal origin) and MSSA isolates were the most diverse. Among the MRSA isolates, the predominant ST were 22, 772, 672, 8 and 30. ST672 is a new emerging clone with only two isolates reported from Australia and U.S. While EMRSA-15 (ST22) appeared as a major clone in Indian hospitals with SCC*mec* type IV element, ST772, 672 and 8 are emerging as SCC*mec* type V. It is evident from our studies that at least two different types of SCC*mec* type V elements exist in isolates belonging to three distinct STs.

The most obvious bias in the study is the limited number of isolates collected, but our results are in part concordant with those in the literature: the two major MRSA STs (STs22 and STs772) reported earlier in India [9,11]. Many of the other MSSA and two of the MRSA STs are being reported for the first time.

The antibiotic sensitivity data (not shown) indicates that majority of carrier MSSA were sensitive to all five tested antibiotics. Antibiotic resistant determinants were found mainly in carrier and disease MRSA isolates, but few ST22 carrier and disease MSSA isolates also had resistance determinants for gentamicin and /or erythromycin. For few MRSA isolates (STs 22, 772, 672, and 8) containing the *mec*A gene, MICs for oxacillin and cefoxitin were 4–8 and 8-16 μg/ml respectively while for most other isolates the corresponding values were 8–16 and 16-32 μg/ml (data not shown). We considered these isolates as methicillin resistant as the patient treatment with oxacillin would select for resistance in a heterogeneous population containing the *mecA* gene. Similar MRSA isolates of ST59 background were found in Taiwan [16] and CC5 lineage in Switzerland among injection drug users. One of the Swiss isolates of CC5 (ZH47) has been reported to have low MIC for oxacillin and sequenced to contain a composite SCC*mec* cassette with ZH47 region containing a second *ccrC*. Our isolates of ST772 and ST672 with low level of oxacillin resistance also contain the second *ccrC* region. The low level of resistance has been attributed to mutations in the *mecA* promoter region [17].

EMRSA-15 (ST22) has been reported to be replacing HA-MRSA in hospitals in many countries - Germany, Portugal, Singapore, to name just a few [18-20]. In 2003 when we had collected MRSA isolates from Indian hospitals [7,8], majority of them belonged to ST239 with SCC*mec* type III or IIIA; ST22 now made up 28% of the total in the present collection. A study from Mumbai, India, with larger sample numbers, from a tertiary care hospital also indicates that EMRSA-15 is replacing type III SCC*mec* containing isolates [11].

ST772 (CC1) has been reported from India, Bangladesh and Malaysia [9,12,13]. Our ST772 isolates and that from Bangladesh have *agr* type II while CC1 isolates from Malaysia, Australia and U.S. have been reported to be *agr* type III. Aires de Sousa et al., have reported three sequence types (ST188, ST573, ST1) belonging to CC1, as *agr* types I, II, and III respectively in a survey of isolates from Portuguese hospitals and community [21]. CC1 lineage itself seems to be changing from an independent founder to a sub-founder and CC15 is evolving as the founder strain from the eBURST analysis (Figure 1). ST573 appears to be the link between the founder and sub-founder clones. CC1 appears to be evolving along with the *agr* locus rapidly with numerous recombinations which is unusual, as *agr* types are usually uniform in a CC.

ST672 has not been reported from any of the Asian countries till now. The MLST data base reports one isolate from Australia and one from U.S. It appears important to determine if this clone will persist as a minor clone or not. ST772 and ST672 MRSA isolates carried the same composite type V SCC*mec* elements unlike the ones carried by ST1208 isolates (Table 2). Among the numerous results obtained by the microarrays, collagen binding adhesion (*cna*) was absent in ST672 and present in 772 (raw data of microarray provided). The capsular polysaccharide types 8 and 5 were present in ST672 and 772 respectively.

The large diversity in the STs present in the MSSA isolates confirmed the highly diverse MSSA population reported from Shanghai, China, recently which included ST5, 6, 7, 30 and 121 isolates along with others [22]. The probability of MSSA conversion to MRSA is perhaps high in India with the over use of antibiotics and its spread due to inadequate hygienic practices.

High prevalence of *PVL* and *egc* among the Indian MSSA and MRSA isolates is unlike the situation in Bangladesh, and Indonesia where only MSSA isolates contain PVL [12,23]. This indicates a possibility of *PVL* positive MSSA acquiring SCC*mec* elements to become *PVL* positive MRSA although this needs to be confirmed. A combination of *PVLegc* along with other entero-toxins could increase the severity of diseases caused by *S. aureus* although the role of *PVL* and other toxins is not completely elucidated [24,25]. There were no differences in the presence of the different virulence factors we characterized among the carrier isolates or the patient isolates.

## Conclusion

This paper reports detailed molecular analysis of *S. aureus* isolates collected from different Indian cities and environments with their virulence factors for the first time. We have identified new and emerging STs as MRSA in addition to already reported ones in healthy carriers as well as patients. There are variant types of type IV and V SCC*mec* elements among MRSA. There is more diversity among the STs found in MSSA which may have the potential to acquire methicillin resistance. Majority of these isolates are *PVL* and *egc* positive. The detailed analysis of virulence factors might help in understanding of diseases caused and influence of host factors in those diseases.

## Methods

### Isolates and patients

Sixty eight *S. aureus* isolates were included in this study, 38 from healthy nasal carriers and 30 from infection sites. Isolates collected from nasal carriers from rural community and urban population between 2006 and 2008 were cultured. Carriers had no identified risk factors for MRSA acquisition which included prior hospitalization, use of antibiotics, and surgeries in the past year. Nasal swabs were collected after explaining the prepared questionnaire and with consent of the subjects. Isolates recovered from infected sites were from wounds, pleural fluid and blood cultures collected in patients from hospitals in Bengaluru, Mumbai, Delhi, and Hyderabad. Data on community origin of these isolates is limited to a few as the isolates were sent to us from physicians from different hospitals. Ethical clearances and written consents for publication were obtained from the respective hospitals.

### Phenotypic characterization

*S. aureus* isolates were selected after growth on chromogenic agar medium (chromAgar, bioMérieux, Marcy-L’Etoile, France) and identified after characterization by Gram staining, detection of catalase, coagulase and DNAse as described elsewhere [26].

### Antibiotic susceptibility testing

Susceptibility testing was performed by Kirby-Bauer disc diffusion according to the guidelines recommended by the CLSI (formerly NCCLS) on Mueller-Hinton agar plates at 37°C using antibiotic discs. Minimum Inhibitory Concentration (MIC) for oxacillin and cefoxitin was determined by the broth dilution method in Mueller-Hinton Broth after 24 hrs of incubation at 37°C in micro titer plates [27].

### Chromosomal DNA isolation

Chromosomal DNA was extracted according to previously published procedures using lysostaphin [7].

### PCR for detection of SCC*mec* elements and *ccr* types

SCC*mec* typing by determination of *mec* and *ccr* complexes for types IV and V SCC*mec* elements was carried out by multiplex PCR [28-30]. Subtyping of type IV SCC*mec* was performed according to the procedure of Zhang et al and Milherico et al [31,32].

### Identification of accessory gene regulator (*agr)* alleles by PCR

The four *agr* alleles were determined by a multiplex PCR as described in Gilot et al [33].

### Detection of toxins

The presence of *PVL* genes was detected by PCR using the published primers and procedure [34]. Presence of staphylococcal entero-toxins A, B, C, D and E, exfoliating toxins A and B and toxic shock syndrome toxin *tst* (TSST-1) and enterotoxin gene cluster (*egc*) cluster were detected by several multiplex PCRs using published procedures [35,36].

### MLST and *spa* typing

MLST and *spa* typing were done as described earlier [37,38].

### PFGE

PFGE was performed as described before [7].

### eBURST analysis

Clonal relationship of the isolates was determined by using eBURST v3 program with the entire MLST database.

### Microarray Analysis using CLONDIAG®

Microarray was performed for selected isolates from each of the clonal complexes. Diagnostic DNA microarray based on the Array/Tube platform (CLONDIAG, Jena, Germany) were utilized as described by Monecke et al [14]. The micro-array covers 185 distinct genes and about 300 alleles there of, including species- specific controls, *agr* alleles, genes including virulence factors, and microbial surface components recognizing adhesive matrix molecules (MSCRAMMS), capsule- type specific genes, as well as resistance determinants and immune evasion factors.

## Authors’ contributions

SS, SN and SP have done the molecular characterization, and helped in organizing tables and figure, MB has planned and executed the microarray, GA has planned the study, executed and drafted the manuscript, JE has helped with microarray and editing the manuscript. All authors have read and approved the manuscript.

## Supplementary Material

Additional file 1 Microarray data: Raw microarray data from 33 isolates representing different STs present in the total of 68 samples. Click here for file

## References

[B1] Chambers HF De Leo FR Waves of resistance:Staphylococcus aureusin the antibiotic era Nat Rev Microbiol 2009 7 629 641 10.1038/nrmicro2200 19680247 PMC2871281

[B2] Feng YC Chen L Su Hu S Yu J Chiu C Evolution and pathogenesis ofStaphylococcus aureus: lessons learned from genotyping and comparative genomics FEMS Microbiol 2008 Rev. 32 23 37 10.1111/j.1574-6976.2007.00086.x 17983441

[B3] Popovich KJ Weinstein RA Hota B Are community associated methicillin-resistantStaphylococcus aureus(MRSA) strains replacing traditional nosocomial MRSA strains? Clin Infect Dis 2008 46 787 794 10.1086/528716 18266611

[B4] Ito T International working group on the classification of Staphylococcal Cassette Chromosome Elements (IWG-SCC) Classification of Staphylococcal cassette chromosomemec(SCCmec): guidelines for reporting novel SCCmecelements Antimicrob Agents Chemother 2009 53 4961 4967 19721075 10.1128/AAC.00579-09 PMC2786320

[B5] Li S Skov RL Han X Larsen AR Larsen J Sorum M Wulf M Voss A Hiramatsu K Ito T Novel types of staphylococcal cassette chromosomemecelements identified in CC398 methicillin resistantStaphylococcus aureusstrains Antimicrob Agents Chemother 2011 55 3046 3050 10.1128/AAC.01475-10 21422209 PMC3101438

[B6] Shore AC Deasy EC Slickers P Brennan G O'Connell B Monecke S Ehricht R Coleman DC Detection of Staphylococcal Cassette ChromosomemecType XI Carrying Highly DivergentmecA, mecI, mecR1, blaZ,andccrGenes in Human Clinical Isolates of Clonal Complex 130 Methicillin-Resistant Staphylococcus aureus Antimicrob Agents Chemother 2011 Aug 55 8 3765 3773 10.1128/AAC.00187-11 21636525 PMC3147645

[B7] Arakere G Nadig S Swedberg G Macaden R Amarnath S Raghunath D Genotyping of methicillin resistantStaphylococcus aureusstrains from two hospitals in Bangalore, South India J Clin Microbiol 2005 43 3198 3202 10.1128/JCM.43.7.3198-3202.2005 16000435 PMC1169171

[B8] Nadig S Namburi P Raghunath D Arakere G Genotyping of methicillin resistantStaphylococcus aureusisolates from Indian Hospitals Curr Sci 2006 91 1364 1369

[B9] Nadig S Sowjanya SV Seetharam S Bharathi K Raghunath D Arakere G Raghunath D, Nagaraja V, Durga Rao C Molecular characterization of Indian methicillin resistantStaphylococcus aureus Proceedings of the Ninth Sir Dorabji Tata Symposium on Antimicrobial resistance-The modern epidemic: Current Status and Research Issues: 10th-11th March 2008 2009 Macmillan 167 184

[B10] Nadig S Ramachandraraju S Arakere G Epidemic methicillin-resistantStaphylococcus aureusvariants detected in healthy and diseased individuals in India J Med Microbiol 2010 59 815 821 10.1099/jmm.0.017632-0 20339016

[B11] D'Souza N Rodrigues C Mehta A Molecular characterization of methicillin-resistantStaphylococcus aureuswith emergence of epidemic clones of sequence type ST 22 and ST 772 in Mumbai India J Clin Microbiol 2010 48 1806 1811 10.1128/JCM.01867-09 PMC2863868 20351212

[B12] Afroz SN Kobayashi S Nagashima MM Alam AB Hossain MA Rahman MR Islam AB Lutfor N Muazzam MA Khan SK Paul AK Shamsuzzaman MC Mahmud AK Mahmud Musa Hossain MA Genetic characterization ofStaphylococcus aureusisolates carrying Panton-Valentine Leukocidin genes in Bangladesh Jpn J Infect Dis 2008 61 393 396 18806351

[B13] Ghaznavi-Rad E Shamsudin MN Sekawi Z Yun Khoon L Nazri Aziz M Hamat RA Othman N Chong PP van Belkum A Ghasemzadeh-Moghaddam H Neela V Predominance and emergence of clones of hospital-acquired methicillin-resistantStaphylococcus aureusin Malaysia J Clin Microbiol 2010 48 867 872 10.1128/JCM.01112-09 20089756 PMC2832418

[B14] Monecke S Slickers P Ehricht R Assignment ofStaphylococcus aureusisolates to clonal complexes based on microarray analysis and pattern recognition FEMS Immunol Med Microbiol 2008 53 237 251 10.1111/j.1574-695X.2008.00426.x 18507678

[B15] Heusser R Ender M Berger-Bachi B McCallum N Mosaic staphylococcal cassette chromosome mec containing two recombinase loci and a new mec complex, B2 Antimicrob Agents Chemother 2007 51 390 393 10.1128/AAC.00921-06 17088487 PMC1797639

[B16] Chen FJK Hiramatsu IW Huang C Wang Lauderdale TL PVL positive methicillin susceptible and resistantStaphylococcus aureusin Taiwan: identification of oxacillin-susceptible mecA positive MRSA Diagn Microbiol Infect Dis 2009 65 351 357 10.1016/j.diagmicrobio.2009.07.024 19766426

[B17] Ender M McCallum N Berger-Bachi B Impact of mecA promoter mutations on mecA expression and beta lactam resistance levels Int J Med Microbiol 2008 298 607 617 10.1016/j.ijmm.2008.01.015 18456552

[B18] Ghebremedhin B Konig W Witte W Hardy KJ Hawkey PM Konig B Subtyping of ST22-MRSA-IV (Barnim epidemic MRSA strain) at a university clinic in Germany from 2002 to 2005 J Med Microbiol 2007 56 365 375 10.1099/jmm.0.46883-0 17314368

[B19] Aires-de-Sousa MB Correia de Lencastre H Multilaboratory Project Collaborators: Changing patterns in frequency of recovery of five methicillin-resistantStaphylococcus aureusclones in portugese hospitals: survelliance over a 16-year period J Clin Microbiol 2008 46 2912 2917 10.1128/JCM.00692-08 18614664 PMC2546730

[B20] Hsu Li-Yang Y Tse-Hsien Koh Kurup A Low J Chlebicki P Ban-Hock Tan High incidence of Panton-Valentine Leukocidin producingStaphylococcus aureusin a tertiary care public hospital in Singapore Clin Infect Dis 2005 40 486 489 10.1086/427033 15668877

[B21] Aires-de-Sousa MT Conceicao C Simas de Lencastre H Comparison of genetic backgrounds of methicillin resistant and susceptibleStaphylococcus aureusisolates from Portuguese hospitals and the community J Clin Microbiol 2005 43 5150 5157 10.1128/JCM.43.10.5150-5157.2005 16207977 PMC1248511

[B22] Han L Ho P Ni Y Zhang H Jiang Y Chu H Sun Y Zhang Y Panton-Valentine Leukocidin-positive MRSA, Shanghai, China Emerg Infect Dis 2010 16 731 733 10.3201/eid1604.081324 20350407 PMC3321931

[B23] Severin JA Lestari ES Kuntaman K Melles DC Pastink M Peeters JK Snijders SV Hadi U Duerink DO van Belkum A Verbrugh HA Antimicrobial Resistance in Indonesia Prevalence and Prevention Study Group Unusually high prevalence of panton-valentine leukocidin genes among methicillin-sensitiveStaphylococcus aureusstrains carried in the Indonesian population J Clin Microbiol 2008 46 1989 1995 10.1128/JCM.01173-07 18434555 PMC2446829

[B24] Labandeira-Rey M Couzon F Boisset S Brown EL Bes M Benito Y Barbu EM Vazquez V Hook M Etienne J Vandenesch F Bowden MG Staphylococcus aureusPanton Valentine Leukocidin causes necrotizing pneumonia Science 2007 315 1130 1133 10.1126/science.1137165 17234914

[B25] Diep BA Palazzolo-Balance AM Tattevin P Basuino L Braughton KR Whitney AR Chen L Kreiswirth BN Otto M Deleo FR Chambers HF Contribution of Panton-Valentine Leukocidin in community-associated methicillin-resistantStaphylococcus aureuspathogenesis PLoS One 2008 3 e3198 10.1371/journal.pone.0003198 18787708 PMC2527530

[B26] Baird D Collee JG, Fraser AG, Marmion BP, Simmons A Staphylococcus: cluster-forming gram positive cocci Practical Medical Microbiology 1996 245 261

[B27] Clinical and Laboratory Standards Institute Performance standards for antimicrobial susceptibility testing: 15th informational supplement 2005 Clinical and Laboratory Standards Institute, Wayne, Pa CLSI/NCCLS document M100-S15

[B28] Oliveira DC de Lencastre H Multiplex PCR strategy for rapid identification of structural types and variants of the mec element in methicillin resistantStaphylococcus aureus Antimicrob Agents Chemother 2002 46 2155 2161 10.1128/AAC.46.7.2155-2161.2002 12069968 PMC127318

[B29] Kondo Y Ito T Ma XX Watanabe S Kreiswirth BN Etienne J Hiramatsu K Combination of multiplex PCRs for staphylococcal cassette chromosome mec type assignment: rapid identification system for mec, ccr, and major differences in junkyard regions Antimicrob Agents Chemother 2007 51 264 274 10.1128/AAC.00165-06 17043114 PMC1797693

[B30] Milheirico C Oliveira DC de Lencastre H Update to the multiplex PCR strategy for the assignment of mec element types in Staphylococcus aureus Antimicrob Agents Chemother 2007 51 3374 3377 10.1128/AAC.00275-07 17576837 PMC2043198

[B31] Zhang K McClure J Elsayed S Louie T Conly JM Novel Multiplex PCR Assay for Characterization and Concomitant Subtyping of Staphylococcal Cassette Chromosome mec Types I to V in Methicillin-Resistant Staphylococcus aureus J Clin Microbiol 2005 43 5026 5033 10.1128/JCM.43.10.5026-5033.2005 16207957 PMC1248471

[B32] Milheirico C Oliveira DC de Lencastre H Multiplex PCR strategy for subtyping the staphylococcal cassette chromosome mec type IV in methicillin-resistant Staphylococcus aureus: ‘SCCmec IV multiplex’ J Antimicrob Chemother 2007 60 42 48 10.1093/jac/dkm112 17468509

[B33] Gilot P Lina G Cochard T Poutrel B Analysis of the genetic variability of genes encoding the RNA III-activating components Agr and TRAP in a population ofStaphylococcus aureusstrains isolated from cows with mastitis J Clin Microbiol 2002 40 4060 4067 10.1128/JCM.40.11.4060-4067.2002 12409375 PMC139642

[B34] Lina G Piemont Y Godail-Gamot F Bes M Peter MO Gauduchon V Vandenesch F Etienne J Involvement of Panton-Valentine leukocidin-producingStaphylococcus aureusin primary skin infections and pneumonia Clin Infect Dis 1999 29 1128 1132 10.1086/313461 10524952

[B35] Mehrotra M Wang G Johnson WM Multiplex PCR for detection of genes forStaphylococcus aureusenterotoxins, exfoliative toxins, toxic shock syndrome toxin1, and methicillin resistance J Clin Microbiol 2000 38 1032 1035 10698991 10.1128/jcm.38.3.1032-1035.2000 PMC86330

[B36] Jarraud S Cozon G Vandenesch F Bes M Etienne J Lina G Involvement of enterotoxins G and I in staphylococcal toxic shock syndrome and staphylococcal scarlet fever J Clin Microbiol 1999 37 2446 2449 10405382 10.1128/jcm.37.8.2446-2449.1999 PMC85251

[B37] Enright MC Day NPJ Davies CE Peacock SJ Spratt BG Multilocus sequence typing for characterization of methicillin resistant and methicillin susceptible clones ofStaphylococcus aureus J Clin Microbiol 2000 38 1008 1015 10698988 10.1128/jcm.38.3.1008-1015.2000 PMC86325

[B38] Shopsin B Gomez M Montgomery SO Smith DH Waddington M Dodge DE Bost DA Riehman M Naidich S Kreiswirth BN Evaluation of protein A gene polymorphic region DNA sequencing for typing ofStaphylococcus aureusstrains J Clin Microbiol 1999 37 3556 3563 10523551 10.1128/jcm.37.11.3556-3563.1999 PMC85690

